# Extra-articular screw placement strategy in Stoppa approach based on three-dimensional reconstruction model

**DOI:** 10.1038/s41598-024-60572-y

**Published:** 2024-04-28

**Authors:** Ruipeng Zhang, Shaojuan Zhang, Xuehong Zheng, Yingchao Yin, Zhongzheng Wang, Siyu Tian, Zhiyong Hou, Yingze Zhang

**Affiliations:** 1 Department of Orthopaedic Surgery, Third Hospital of Hebei Medical University, Shijiazhuang, China; 2 Department of Cardiology, Second Hospital of Hebei Medical University, Shijiazhuang, China; 3 Department of Orthopaedic Surgery, Hebei General Hospital, Shijiazhuang, China

**Keywords:** Outcomes research, Preclinical research, Trauma

## Abstract

The study aimed to explore an extra-articular screw placement strategy in Stoppa approach. Radiographic data of patients who underwent pelvic computed tomography from January 2016 to June 2017 were imported into Materiaise’s interactive medical image control system software for three-dimensional reconstruction. Superior and lower margins of acetabulum and ipsilateral pelvic brim could be observed simultaneously through inlet-obturator view. A horizontal line from superior acetabular margin intersected pelvic brim at point “A” and another vertical line from lower margin intersected pelvic brim at point “B” were drawn, respectively. Lengths form sacroiliac joint to “A” (*a*), “A” to “B” (*b*), and “B” to pubic symphysis (*c*) were measured. Patients were divided into four groups depending on gender and side difference of measured hemi-pelvis: male left, male right, female left, and female right. Lengths of adjacent holes (*d*) and spanning different holes (*e*) of different plates were also measured. Mean lengths of *a, b, c* in four groups were 40.94 ± 1.85 mm, 40.09 ± 1.93 mm, 41.78 ± 3.62 mm, and 39.77 ± 2.23 mm (*P* = 0.078); 40.65 ± 1.58 mm, 41.48 ± 1.64 mm, 40.40 ± 1.96 mm, and 40.66 ± 1.70 mm (*P* = 0.265); 57.03 ± 3.41 mm, 57.51 ± 3.71 mm, 57.84 ± 4.40 mm, and 59.84 ± 4.35 mm (*P* = 0.165), respectively. Mean *d* length of different plates was 12.23 mm. Average lengths spanning 1, 2, 3 and 4 holes were 19.33 mm, 31.58 mm, 43.80 mm, and 55.93 mm. Our data showed that zones *a* and *c* could be safely inserted three and four screws. Penetration into hip joint could be avoided when vacant 3-hole drilling was conducted in zone *b*. Fracture line in zone *b* could serve as a landmark for screw placement.

## Introduction

It was reported that anatomic reduction and rigid fixation could improve the functional and clinical results for pelvic or acetabular fractures^1,2^. Both ilioinguinal and Stoppa approaches were frequently used anterior techniques for the injury of this area^3,4^. Compared with ilioinguinal approach, a less invasive incision with lower blood loss may be accompanied by Stoppa procedure^5^. What’s more, area from the pubic symphysis to the bilateral sacroiliac joints including quadrilateral surface could be directly exposed through Stoppa approach^3,6–8^. Then, Stoppa technique has been widely applied to treat pelvic and acetabular fractures since it was proposed^2,6,9^. However, penetration into hip joint may occur during the drilling procedure because direct visualization of articular surface could not be obtained through the Stoppa window, which would damage articular cartilage result in serious clinical consequences^9,10^. To avoid screw misplacement into joint cavity in Stoppa technique, repeated intraoperative fluoroscopy and adjustment of screws were needed^11^. However, it was time consuming.

Several studies had been performed to accomplish the extra-articular screw placement in Stoppa approach^10,12,13^. Guy P et al. reported the safe distances for screw placement to femoral head, sciatic notch and obturator canal based on CT scans, nevertheless, relevant structures were difficult to expose directly during surgical process^10^. Ji et al. reported the ranges of safe insertion angles and maximum screw lengths in five different sections through digital anatomical measurements, nevertheless, it was challenging to precisely locate the specific section intraoperatively because there was no obvious landmark in surgical procedures^12^. Three-dimension navigation has been used in screw insertion for anterior pelvic fractures. However, preoperative preparation was time consuming and non-displacement of surgical site was required during drilling, which may restrict its clinical application^13^. What’s more, relevant device was so expensive that many primary hospitals could not bear the fiscal burden.

Three-dimensional reconstruction and relevant measurement could be achieved by Materiaise’s interactive medical image control system (MIMICS) software, which has been employed in clinical orthopedics^14,15^. The study aimed to explore an extra-articular screw placement strategy in Stoppa approach based on three-dimensional reconstruction model through MIMICS software.

## Methods

### Data collection

Patients who underwent CT scans in pelvis from January 2016 to June 2017 were retrospectively analyzed in this study. The inclusion criteria were as follows: 18–65 years old, with normal anatomy in pelvis before injury, unilateral pelvic or acetabular injury, with intact preoperative CT data of pelvis. The exclusion criteria of patients were as follows: pathologic fractures, pelvic malformation, with pelvic or celiac disease, inadequate preoperative CT data of pelvis. A total of 80 patients (including 34 patients of acetabular fractures and 46 cases of pelvic ring injury) with an average age 43.78 years were included in this study. They were divided into four groups depending on gender and side difference of measured hemi-pelvis: male left (ML), male right (MR), female left (FL), and female right (FR). There were 28 cases of group ML, 17 cases of group MR, 20 cases of group FL, and 15 cases of group FR, respectively. All preoperative CT scans were conducted by a 128-slice Siemens CT scan (GE) with 1.0 mm slices at 0.1-s intervals for imaging of the pelvis. The raw data were stored in DICOM (Digital Imaging and Communications in Medicine) format in this study. All methods were performed in accordance with the relevant guidelines and regulations in surgery (STROCSS) criteria^16^. Institutional Review Board of Third Hospital of Hebei Medical University approved the study and waived informed consent from all the patients and/or their legal guardian(s).

### Model reconstruction and measurement

**Figure 1:**
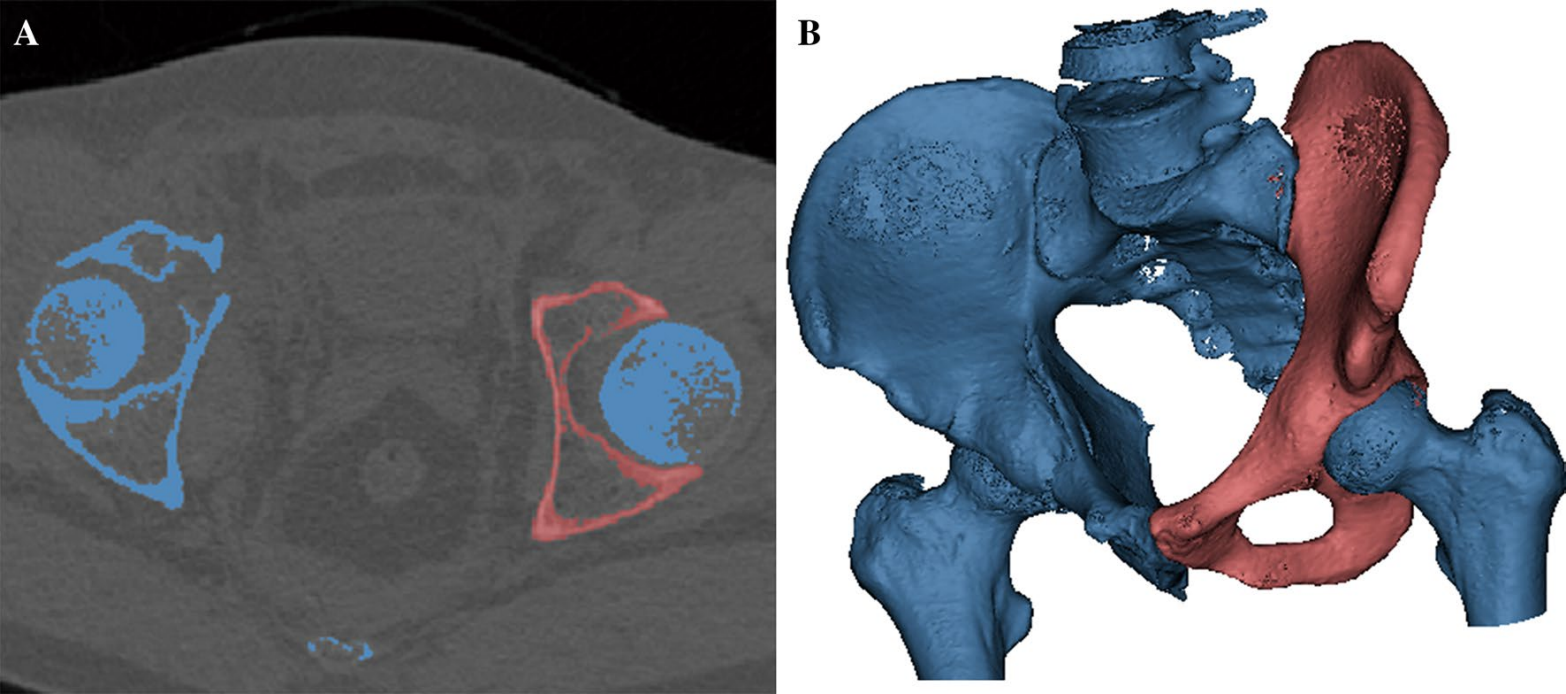
Three-dimensional reconstruction model of pelvis was obtained by MIMICS software. (**A**) Uninjured hemi-pelvis and other parts of pelvis were marked with red and blue colors, respectively; (**B**) three-dimension reconstruction inlet-obturator view of uninjured hemi-pelvis.

Relevant raw data were imported into MIMICS 20.0 software (Materialise, Leuven, Belgium) for three-dimensional reconstruction. Uninjured hemi-pelvis and other bony structures (including sacrum, the affected hempelvis and bilateral femoral heads) were differently marked, respectively (Fig. 1A). The reconstructed uninjured hemi-pelvis model was rotated to inlet-obturator view to obtain direct visualization of the maximum diameter of acetabulum and ipsilateral pelvic brim simultaneously (Fig. 1B).

The transparency of software was adjusted to clearly reveal the superior and lower margins of acetabulum. A horizontal line (with 3.5 mm diameter, representing drilling trajectory) from superior margin was drawn, which intersected pelvic brim at point “A” (how to locate point “A” was presented as below). The horizontal connection line between point A and acetabular upper edge located in cross section. The cross section, perpendicular to the major axis of the human body, passing the upper edge of the acetabulum was specific. This specific cross section did not vary with the rotation of the pelvic model. Then, the position of point A, the intersection of pelvic brim and the specific cross section, was unique.

Thereafter, a vertical line from lower margin was drawn, which intersected pelvic brim at point “B” (how to locate point “B” was presented as below). Regarding pelvic brim (from the symphysis pubis to the sacroiliac joint) as a portion of a circle, the specific center could be conformed in MIMICS software. This position of circle center did not vary with the rotation of the pelvic model. The specific line, connecting circle center and the lower edge of acetabulum, was regarded as perpendicular to circle (pelvic brim). Then, the position of point B, the intersection of pelvic brim and specific line (blue dotted line) was unique.

**Figure 2:**
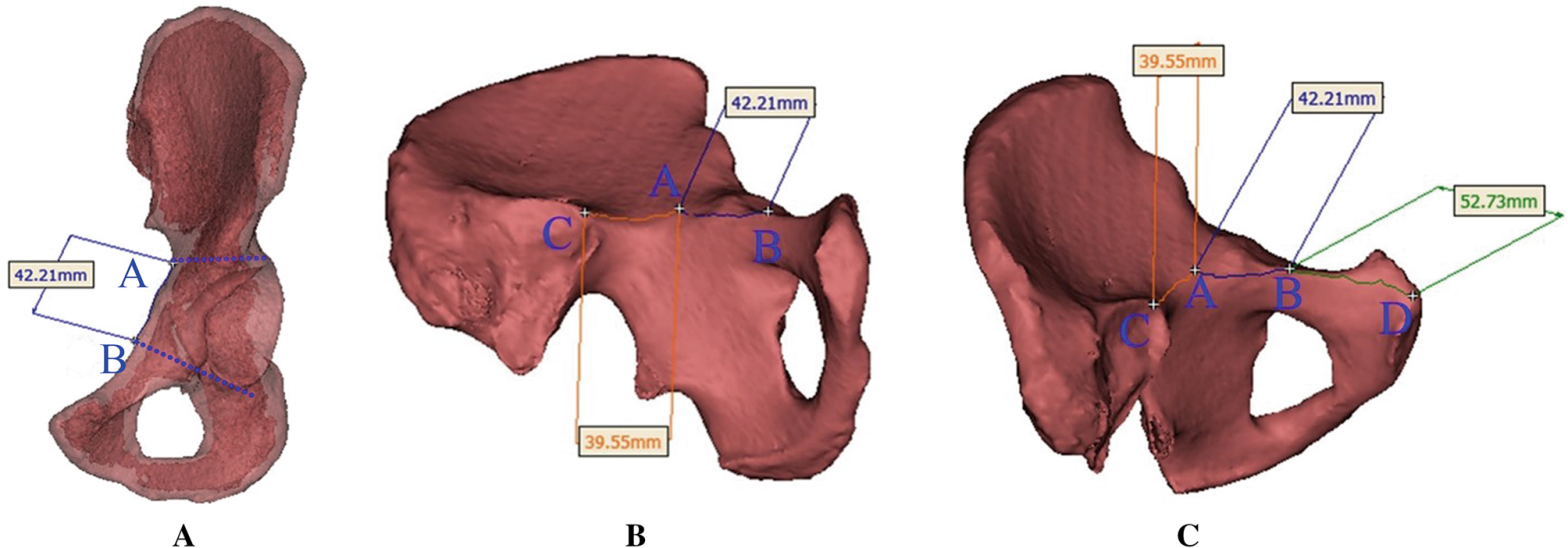
Lengths of three zones were measured successively by surface distance measurement function of MIMICS software. (**A**) Length measurement of *b*; (**B**) length measurement of *a*; (**C**) length measurement of *c*.

Length from point “A” to “B” (*b*) was measured by surface distance measurement function of MIMICS software. The reconstructed model was rotated successively to expose the sacroiliac joint (point “C”) and pubic symphysis (point “D”) in pelvic brim. The surface distances from “A” to “C” (*a*) and “B” to “D” (*c*) could also be measured by the software (Fig. 2).

**Figure 3:**
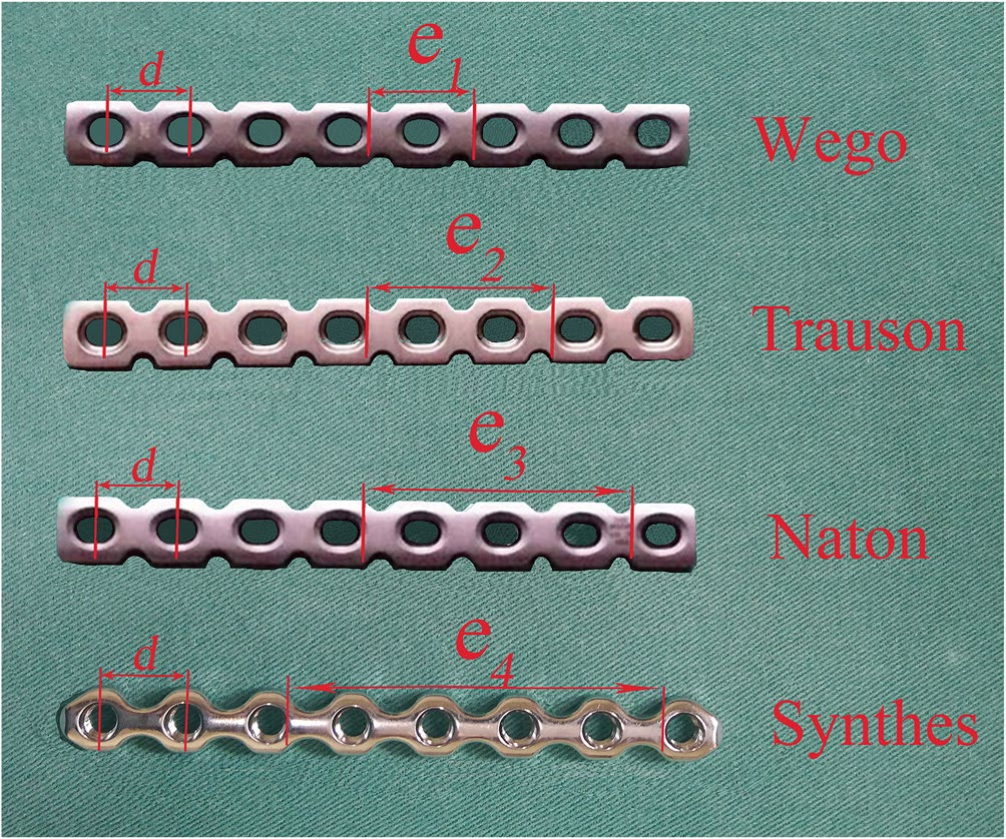
Distances of adjacent holes (*d*) and lengths spanning one, two, three, and four holes (*e*_1_, *e*_2_, *e*_3_, and *e*_4_) of different plates were measured.

Reconstruction plates of different manufacturers including Wogo, Trauson, Naton and Synthes were included in this study. For different plates, lengths of two adjacent holes (*d*), spanning one, two, three, and four holes (*e*_1_, *e*_2_, *e*_3_, and *e*_4_) were measured successively (Fig. 3). Relevant measurement of uninjured hemi-pelvis (*a*, *b*, *c*) and different reconstruction plates (*d*, *e*) were verified by two investigators. And the average of measurement of two investigators was regarded as the research data in this study to reduce the impact of measurement errors. Extra-articular screw placement strategy in Stoppa approach could be determined based on the relevant data of uninjured hemi-pelvis model and reconstruction plates.

### Definition of the dangerous and safe zone

Area from point “A” to “C” located in the superior part of pelvic brim in the supine position. It was difficult to drill caudad in this region because of the block of abdominal organs, especially for the fat patients^9^. Screws were usually inserted vertically to bone surface (or mildly cephalad) in zone *a* clinically, whose trajectory was farther away from the hip joint than horizontal line drawn in the MIMICS software. Then, screws inserted in zone *a* through Stoppa approach would not penetrate into acetabular surface. Area from point “B” to “D” was the lower portion of pelvic brim. Cephalad drilling was difficult to conduct in zone *c* because of the limitation of Stoppa surgical incision and the special anatomy of inner surface in pelvic brim. Clinically, screws were usually placed vertically or caudad in zone *c*, whose trajectory was also farther away from the hip joint than vertical line. Drilling performed in zone *c* through Stoppa approach would not penetrate into hip joint, either. Thus, *a* and *c* were defined as safe zones for extra-articular screw placement. However, area from point “A” to point “B” was the inner surface of acetabular socket. Drilling procedure in this region (zone *b*) was technically demanding and high incidence of intra-articular screw placement may be accompanied. Then, *b* was regarded as dangerous zone in this study.

### Statistical analysis

The statistical data in this study were processed using SPSS (version 23.0; SPSS, Chicago, IL). Relevant data of this study were expressed as mean ± standard deviation. *T*-test and one-way anova were employed to analyze two-sample and multi-group data, respectively. A value of *P* < 0.05 was considered statistically different in this study.

## Results

Mean lengths of male and female pelvic brim were 138.80 ± 4.46 mm and 140.13 ± 7.39 mm, respectively (*P* = 0.321). Average lengths of pelvic brim in ML and MR groups were 138.63 ± 4.29 mm and 139.08 ± 4.86 mm, respectively (*P* = 0.745). Mean lengths of pelvic brim in FL and FR were 140.02 ± 8.44 mm and 140.27 ± 5.99 mm, (*P* = 0.921). Mean lengths of zone *a*,* b*, and* c* in four groups (ML, MR, FL, and FR) showed no statistical difference in this study (Table 1). Mean *d* length of plates from different manufacturers was 12.23 mm in this study. Average lengths spanning 1, 2, 3 and 4 holes in different plates were 19.33mm, 31.58mm, 43.80mm, and 55.93mm, respectively (Table 2). Area *a* and *c* were defined as safe zones for extra-articular screw placement in this study, then, plate with screw fixation located in those regions should not exceed their respective lengths*.* The number (n) of screws not penetrated into hip joint could be calculated according to the formula: n*d* ≤ *a* and n*d* ≤ *c.* However, Area *b* was defined as dangerous zone in this study. Plate without drilling in this region should not be less than the length of zone *b* (*e*_*n*_ ≥ *b*). Therefore, three and four screws could be inserted in zone *a* and *c* according to calculation, respectively. However, three holes of plate should be empty in zone *b* to avoid intra-articular screw placement during Stoppa technique. It was summarized as the concise 3/3/4 screw placement principle in zone *a/b/c* in this study.

**Table 1:** Average lengths of the *a*, *b* and *c* regions in four groups.

Group	*a* (mm)	*b* (mm)	*c* (mm)
ML	40.94 ± 1.85	40.65 ± 1.58	57.03 ± 3.41
MR	40.09 ± 1.93	41.48 ± 1.64	57.51 ± 3.71
FL	41.78 ± 3.62	40.40 ± 1.96	57.84 ± 4.40
FR	39.77 ± 2.23	40.66 ± 1.70	59.84 ± 4.35
*P*	0.078	0.265	0.165

Average lengths of the *a*, *b* and *c* regions of four groups showed no statistical difference.

**Table 2:** Lengths of *d* and *e* from different manufacturers.

Manufacturers	*d* (mm)	*e*_1_ (mm)	*e*_2_ (mm)	*e*_3_ (mm)	*e*_4_ (mm)
Wego	12.1	19.1	31.1	43.3	55.2
Trauson	12.1	19.2	31.2	43.5	55.4
Naton	12.2	19.2	31.4	43.2	55.3
Synthes	12.5	19.8	32.6	45.2	57.8
Mean	12.23	19.33	31.58	43.80	55.93

Lengths of two adjacent holes (*d*), spanning one, two, three, and four holes (*e*_1_, *e*_2_, *e*_3_, and *e*_4_) were presented.

## Discussion

Pelvic brim (from the symphysis pubis to the sacroiliac joint) was curved and its arc length is difficult to measure in reality. Lengths of *a, b*, and *c* in pelvic brim were measured in this study. Results of this study demonstrated that zone *a* and *c* could be safely inserted three and four screws through Stoppa approach, respectively. However, drilling penetration into hip joint could be avoided when vacant 3-hole drilling in zone *b*.

Stoppa approach, as a minimally invasive technique, has been widely employed to manage pelvic and acetabular fractures^6,8,17^. Fragments of quadrilateral surface in acetabular fractures were frequently medially displaced with the strike from femoral head, then, plate should be placed at the inner surface of pelvic brim in Stoppa procedure to provide blocking effect for displaced fragment^2,6,9^. However, extra-articular screw placement was a challenging issue in Stoppa approach because euthyphoria of the hip joint was impossible through the minimally invasive incision^10,12,14^.

To accomplish the extra-articular drilling in Stoppa technique, three-dimensional pelvic model reconstructed by MIMICS software was applied in this study (Fig. 1). The anterior boundary of pelvic brim (area for plate placement) and the maximum diameter of acetabular socket could be simultaneously revealed through inlet-obturator view. Then, it was employed to determine the superior and lower margins of acetabulum in this study. Clinically, the diameter of inserted screw in pelvic or acetabular surgery was 3.5 mm^9,18^. Thus, dotted lines with the same diameter were employed in the three-dimensional reconstruction model (Fig. 2A) to represent the drilling trajectory in Stoppa approach. The three-dimensional model was obtained through MIMICS software based on the CT raw data in this study, which was a 1:1 reconstruction of pelvis. A curve measuring tool, surface distance measurement in MIMICS software, was employed to gain accurate data of three zones in hemi-pelvic brim. Results revealed a fact that there was no statistical difference in the lengths of the three zones in hemi-pelvic brim for patients of different genders and sides. It was also demonstrated that length characteristics were similar in reconstruction plates from different companies. Therefore, it was possible to formulate the extra-articular screw placement strategy in the Stoppa approach based on the length characteristics of the pelvic brim and plates.

A 7–10 holes plate was usually recommended through Stoppa incision to obtain adequate stability of displaced fragments in pelvic or acetabular fractures. However, standard plate placement for every patient was difficult to accomplish because there was no anatomic landmark. It was recommended that plate be placed in the inner surface of quadrilateral surface and its anterior edge be aligned with pelvic brim during the surgical procedure to achieve a more normative plate placement. Zone *b*, as inner surface of acetabulum in pelvic brim, screw insertion was not recommended to avoid penetration into hip joint. It was not practical to place all screws vertically to bone surface of pelvic brim through Stoppa approach because of limited working space, especially for fat patient. The use of a large retractor to pull the contents of the abdominal cavity to the contralateral side of the fracture, combined with the application of a protective nail sleeve may help perpendicular drilling to the bone surface. Actually, there was no obvious definite landmark of “A” and “B” during Stoppa surgical procedure. However, distances from point “A” to “B” (b), “A” to “C” (a) and “B” to “D” (c) were measured through MIMICS software. Results showed that mean lengths of zone *a, b*, and *c* (almost 4cm, 4cm, and 6cm) in four groups (ML, MR, FL, and FR) showed no statistical difference in this study. The symphysis pubis and the sacroiliac joint served as landmark, combined with measurement result of this study, could guide plate and screw placement in Stoppa procedure. For example, taking symphysis pubis as a landmark on pelvic brim, penetration into hip joint could be easily avoided when screws were placed in the range 0–6 cm and 10–14 cm from symphysis pubis. Drilling within 6–10 cm from symphysis pubis was not recommended to avoid screw penetration.

Several studies have been performed in past decades to avoid intra-articular screw placement through Stoppa window, however, absence of obvious anatomic landmark and relatively complicated results may restrict their clinical generalization^9,10,14^. Zhang et al. first reported that the insertion of the psoas minor tendon at the pelvic brim (IPMTPB) could serve as an anatomic landmark for extra-articular screw placement in Stoppa window^9^. However, psoas minor was presented in only 53.33% specimens and the IPMTPB may be destroyed during fracture or surgical exposure. To guide the drilling procedure for the patients without psoas minor, relevant lengths of three zones divided by IPMTPB were measured through a tape. Certain deviation of the results may be accompanied because the curved pelvic brim was measured by a linear measuring tool in that study. Compared with his study, more accurate data could be gained through the curve measuring tool proposed by this study. Nevertheless, the curvature in each zone was small or negligible, then, relevant data of three zones divided by IPMTPB was similar to that obtained through three-dimensional model. It also demonstrated that the IPMTPB could serve as an anatomic landmark for extra-articular drilling in Stoppa technique. What’s more, the concise 3/3/4 screw placement principle in zone *a/b/c* was proposed in this study. Intraoperative complicated measurement process required by previous studies was unnecessary, which would greatly lower the operation time.

**Figure 4:**
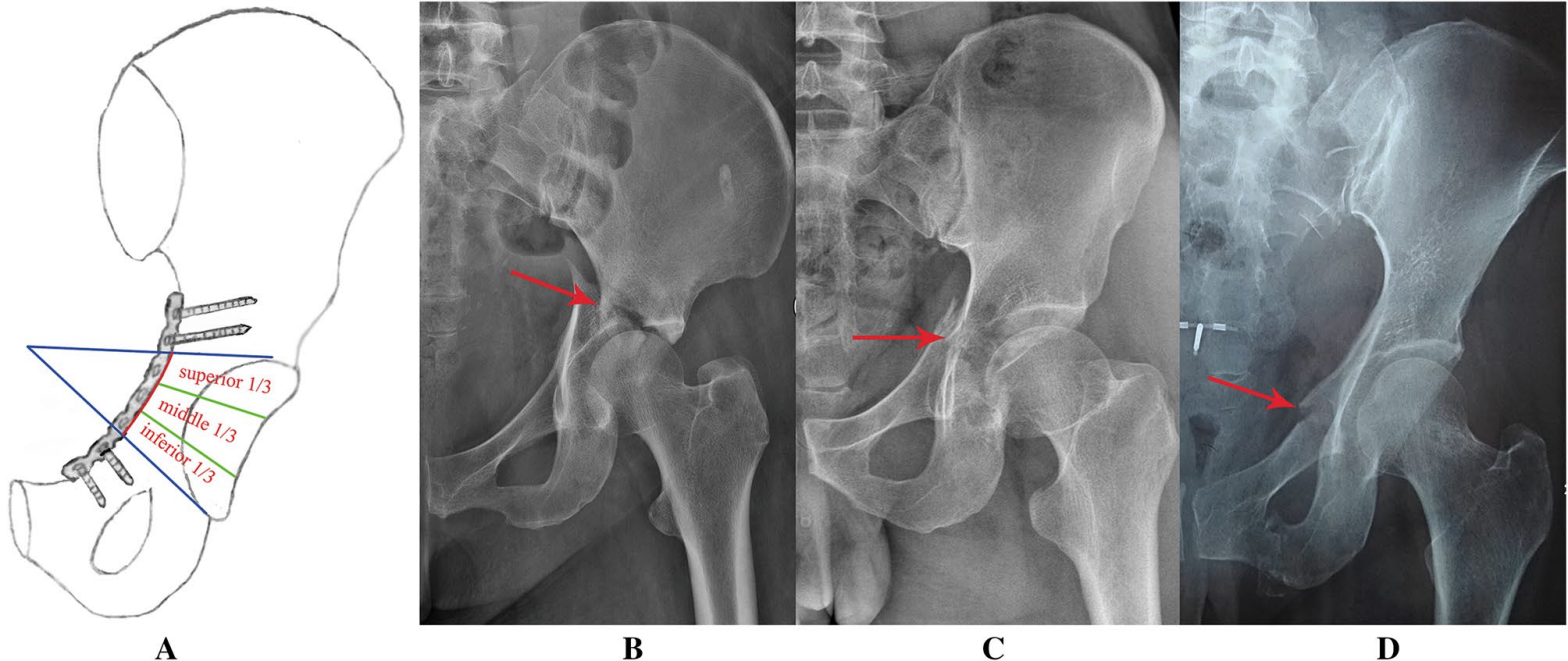
Zone *b* was further divided equally into three parts to accomplish extra-articular screw placement for patients of acetabular fractures. (**A**) Schematic diagram of three parts in zone *b* and plating technique in Stoppa approach; (**B**) fracture line in superior 1/3 of zone *b;* (**C**) fracture line in middle 1/3 of zone *b;* (**D**) fracture line in inferior 1/3 of zone *b*.

**Figure 5:**
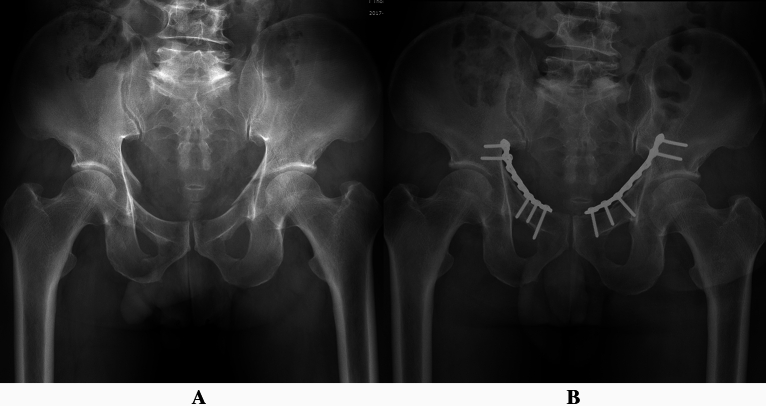
Preliminary clinical application of the technique proposed in this study. (**A**) Preoperative anteroposterior radiograph of a male patient with bilateral acetabular fractures; (**B**) extra-articular screw placement was accomplished based on the results obtained in this study.

For patients of acetabular fractures, it was difficult to determine the plating position based on the location of fracture line. However, fracture line in the pelvic brim, visible after the reduction of quadrilateral surface fragment, could be regarded as a landmark for plate placement through the Stoppa window. Zone *b* (dangerous zone) was further divided equally into three parts based on the length characteristics of pelvic brim and reconstruction plates (Fig. 4). A reference hole not drilling was aligned with the fracture line in pelvic brim. For fracture line in superior 1/3 of zone *b*, drilling in distally adjacent two holes (relative to the reference hole) was not recommended. For the fracture line in middle 1/3 of zone *b*, insertion was not recommended in proximal and distal first adjacent holes (relative to the reference hole). For the fracture line in inferior 1/3 of zone *b*, drilling in proximally adjacent two holes (relative to the reference hole) was not recommended (Fig. 4). Preliminary clinical practice proved that extra-articular screw placement through Stoppa approach could be accomplished with the drilling technique (Fig. 5).

There were some limitations in this study. The limited patient number was a drawback of this study. Relevant lengths of plate holes and hole direction after pre-countering procedures may be inconsistent with that measured on straight plates. However, its change was relatively minimal, and even negligible. What’s more, the concise 3/3/4 screw placement principle in three zones had been tested in clinic and satisfactory result were obtained. Only four kinds of plates were involved in this study, which may not represent features of all reconstruction plates. Then, 3/3/4 screw placement principle in zone *a/b/c* may not be appropriate for all reconstruction plates. Clinical trials including more cases and plates should be performed in the future to further validate extra-articular screw placement strategy in Stoppa approach.

## Conclusions

Zones *a* and *c* could be safely inserted three and four screws, respectively. Penetration into hip joint could be avoided when vacant 3-hole drilling was conducted in zone *b*. The concise 3/3/4 screw placement principle in zone *a/b/c* of hemi-pelvic brim was an alternative drilling technique in Stoppa window. For acetabular fractures, fracture line in zone *b* could be regarded as a reference for plate and screw placement in Stoppa approach.

## Data Availability

The datasets used and analyzed during the current study available from the corresponding author on reasonable request.
